# Secretory expression of β-1,3-glucomannanase in the oleaginous yeast *Rhodosporidium toruloides* for improved lipid extraction

**DOI:** 10.1186/s40643-023-00639-2

**Published:** 2023-03-02

**Authors:** Shiyu Liang, Yue Zhang, Liting Lyu, Shuang Wang, Zongbao K. Zhao

**Affiliations:** 1 Laboratory of Biotechnology, Dalian Institute of Chemical Physics, CAS, 457 Zhongshan Road, Dalian, 116023 China; 2 University of Chinese Academy of Sciences, Beijing, 100049 China; 3 Dalian Key Laboratory of Energy Biotechnology, Dalian Institute of Chemical Physics, CAS, Dalian, 116023 China

**Keywords:** Cell wall engineering, β-1,3-glucomannanase, Lipid extraction, Oleaginous yeast, *Rhodosporidium toruloides*, Secretory expression

## Abstract

Lipids produced by oleaginous yeasts are considered as sustainable sources for the production of biofuels and oleochemicals. The red yeast *Rhodosporidium toruloides* can accumulate lipids to over 70% of its dry cell mass. To facilitate lipid extraction, a recombinant β-1,3-glucomannanase, MAN5C, has been applied to partially breakdown *R. toruloides* cell wall. In this study, *R. toruloides* NP11 was engineered for secretory expression of MAN5C to simplify the lipid extraction process. Specifically, a cassette contained a codon-optimized gene *MAN5C* was integrated into the genome of *R. toruloides* by *Agrobacterium*-mediated transformation. The engineered strain NP11-MAN5C was found with proper expression and secretion of active MAN5C, yet no notable compromise in terms of cell growth and lipid production. When NP11-MAN5C cell cultures were extracted with ethyl acetate without any pretreatment, 20% of total lipids were recovered, 4.3-fold higher than that of the parental strain NP11. When the cells were heat-treated followed by extraction with ethyl acetate in the presence of the culture broth supernatants, up to 93% of total lipids were recovered, confirming beneficial effects of MAN5C produced in situ. This study provides a new strategy to engineer oleaginous yeasts for more viable lipid extraction and down-stream processes.

**Graphical Abstract:**

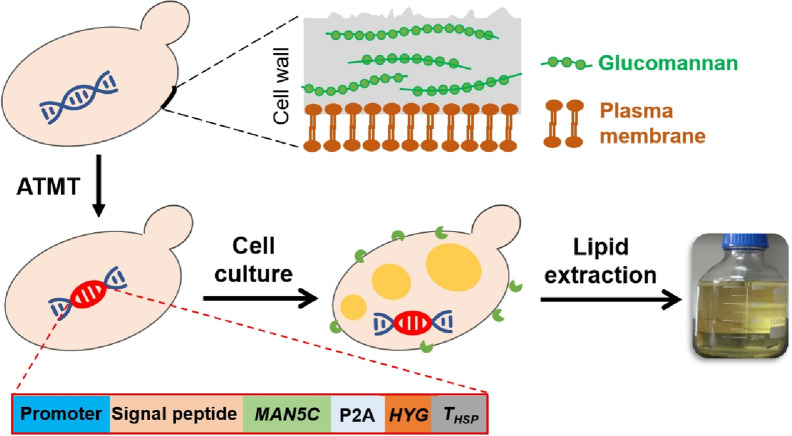

**Supplementary Information:**

The online version contains supplementary material available at 10.1186/s40643-023-00639-2.

## Introduction

The basidiomycetous yeast *Rhodosporidium toruloides*, now classified as *Rhodotorula toruloides*, is oleaginous, carotenogenic and of potential biotechnological significance, as it can accumulate lipids to over 70% of its dry cell weight (DCW) under nutrient-limited conditions, and achieve high cell density when cultivated in a stirred-tank bioreactor (Li et al. 2007; Wen et al. 2020). Thus, diverse oleochemicals and terpenoids have been produced by as-isolated or genetic engineered *R. toruloides* cells (Jin et al. 2013; Jiao et al. 2018; Liu et al. 2020, 2021a; Zhang et al. 2021, 2022). While natural *R. toruloides* strains can readily utilize a wide range of low-cost carbon sources and resist to some toxic compounds found in the biomass hydrolysates (Hu et al. 2009; Park et al. 2018), further improvements in terms of robustness have been documented via adaptive laboratory evolution and genetic engineering (Liu et al. 2021b; Lyu et al. 2021). Therefore, *R. toruloides* is promising for the production of lipids from low-cost substrates.

**Fig. 1:**
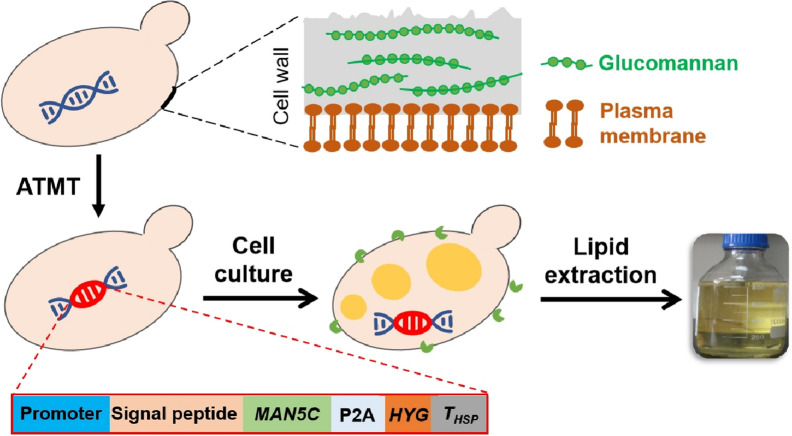
Diagram of the strategy for expression of the cell wall lytic enzyme MAN5C in *R. toruloides* for improved lipid extraction

However, downstream processes for the recovery of yeast lipids remain costly and laborious, especially for large-scale operations (Dong et al. 2016). Yeast cells are wrapped with thick cell wall that prevents leaking of intracellular metabolites and hinders organic solvents for the extraction of hydrophobic products. To facilitate lipid extraction, diverse pretreatment processes have been described to breakdown the cell wall after cultivation, as the composition and structure of cell wall can be quite different among different species (Bonturi et al. 2015; Bzducha-Wrobel et al. 2013; Zainuddin et al. 2021). The cell wall of *R. toruloides* composes mainly of glucomannan and chitin (Fig. 1), such that it is resistant to common cell wall lytic enzymes including glucanase (Arai et al. 1978; Murao et al. 1976). An enzyme named MAN5C with β-1,3-glucomannanase activity firstly purified from the fungi *Penicillium lilacinus* ATCC 36010, was able to damage the cell wall of *R. toruloides* to form protoplast (Murao et al. 1976; Yang et al. 2011). The application of this enzyme led to the development of a simple colony PCR method for red yeast (Lin et al. 2012) and an enzyme-assisted approach for lipid extraction from the cell culture of *R. toruloides* (Jin et al. 2012). Remarkably, *R. toruloides* cells treated with microwave irradiation followed by MAN5C led to the collapse of cell wall surface and total lipid recovery yield of 96% by extraction with ethyl acetate. Compared with other methods such as strong acid or base treatment, bead milling, high-speed homogenization, ultrasonication and hydrothermal liquefaction (Zainuddin et al. 2021), enzyme-assisted lipid extraction required less equipment investment and lower energy consumption. However, the preparation of MAN5C as an agent would increase the total costs.

Cell wall engineering is a potential way to improve its digestibility and related properties. Modification of plant cell walls have been described previously by the expression of enzymes capable of hydrolyzing polysaccharides or consuming the precursors for lignin biosynthesis to improve biomass hydrolysis (Eudes et al. 2015; Hao et al. 2021; Pogorelko et al. 2011), however, similar approaches have not been adopted to weaken the cell wall of oleaginous yeasts. It has been suggested that the expression of genes coding for glucanase, chitinase, and similar hydrolytic enzymes may alter the cell wall structure, thus facilitate cell recovery and lipid extraction (Khot et al. 2020). On the other hand, alteration of the cell wall integrity may lead to reduced cell growth and compromised lipid production capacity. Thus, it remains challenging to balance the merits of the expression of a lytic enzyme for lipid extraction and the demerits on cell physiology related to lipid production. Here, we reported the results of engineering *R. toruloides* with secretory expression of MAN5C to enhance lipid extraction (Fig. 1). Specifically, a cassette contained the codon-optimized gene *MAN5C* was integrated into the genome of *R. toruloides* NP11 by *Agrobacterium*-mediated transformation (ATMT), leading to an engineered strain NP11-MAN5C. Results showed that NP11-MAN5C cells retained good lipid production profiles and could secrete active MAN5C into the culture media. Lipid extraction from NP11-MAN5C cells with ethyl acetate was greatly improved by using MAN5C produced in situ. This study provides a new strategy to engineer yeasts for more practical extraction of intracellular products and down-stream processes.

## Materials and methods

### Strains, media and reagents

Strains used in this study are listed in Table 1. *Rhodosporidium toruloides* NP11 (GDMCC 2.224) is a haploid strain separated from *R. toruloides* CGMCC 2.1389 by our laboratory (Zhu et al. 2012). *Escherichia coli* DH10B was used for plasmid construction. *Agrobacterium tumefaciens* AGL1 was use for transformation experiments.

**Table 1:** Strains and plasmids used in this study

Strains or plasmids	Genotype or characteristic	Resource
Strains		
4#	*R. toruloides* CGMCC 2.1389	CGMCC
NP11	*R. toruloides* GDMCC 2.224, *MAT A1*	(Zhu et al. 2012)
*Escherichia coli* DH10B	*F- endA1 deoR recA1 galE15 galK16 nupG, rpsLΔ(lac)X74 Φ80lacZΔM15 araD139 Δ(ara,leu)7697 mcrA Δ(mrr-hsdRMS-mcrBC)* Str^R^ λ^−^	Invitrogen
*Agrobacterium tumefaciens* AGL1	*AGL0 recA::bla pTiBo542DT Mop* + *CbR*	(Lin et al. 2014)
*P. pastoris* pPICZαA-plman5c	*P. pastoris* X-33 carrying the plasmid pPICZαA-plman5c	(Yang et al. 2011)
NP11-MAN5C	NP11 carrying the P_*ADH2*_-*MAN5C*-P2A-*HYG*-T_*HSP*_ expression cassette	This study
Plasmid		
pZPK-P_*ADH2*_	P_*ADH2*_ in pZPK	Lab collection
pZPK-P_*PGK*_-*MNP*-P2A-*HYG*-T_*HSP*_	P_*PGK*_-*MNP*-P2A-*HYG*-T_*HSP*_ in pZPK	(Lyu et al. 2021)
pUC-MAN5C	*MAN5C* in pUC57	This study
pZPK-P_*PGK*_-*MAN5C*-P2A-*HYG*-T_*HSP*_	P_*PGK*_-*MAN5C*-P2A-*HYG*-T_*HSP*_ in pZPK	This study
pZPK-P_*ADH2*_-*MAN5C*-P2A-*HYG*-T_*HSP*_	P_*ADH2*_-*MAN5C*-P2A-*HYG*-T_*HSP*_ in pZPK	This study

*R. toruloides* strains were cultured at a rotary rate of 200 rpm at 30 ℃ in yeast extract-peptone-dextrose (YEPD) medium (10 g/L yeast extract, 20 g/L peptone, 20 g/L glucose, pH 6.0). Nitrogen-limited (NL) medium (50 g/L glucose, 0.1 g/L (NH_4_)_2_SO_4_, 0.75 g/L yeast extract, 1 g/L KH_2_PO_4_, 1.5 g/L Mg_2_SO_4_·7H_2_O, 10 mL/L trace element stock, 100 μL/L 18 M H_2_SO_4_; trace element stock contained 4.0 g/L CaCl_2_·2H_2_O, 0.55 g/L FeSO_4_·7H_2_O, 0.52 g/L citric acid monohydrate, 0.1 g/L ZnSO_4_·7H_2_O, 0.076 g/L MnSO_4_·7H_2_O) was used for lipid production. *E. coli* and *A. tumefaciens* were cultured in Luria–Bertani (LB) medium (5.0 g/L yeast extract, 10 g/L tryptone, 10 g/L NaCl) at 37 ℃ and 30 ℃, respectively. Induction medium was prepared as described (Lin et al. 2014). All of the plates were added 15 g/L agar into the corresponding liquid medium. Antibiotics were supplemented to the medium to a final concentration as following: 50 μg/mL kanamycin, 100 μg/mL ampicillin, 50 μg/mL hygromycin, and 300 μg/mL cefotaxime.

Yeast extract and tryptone were purchased from Oxoid (Basingstoke, UK). Peptone was purchased from BD Difco (Thermo Fisher Scientific, USA). Antibiotics, acetosyringone and agar powder were supplied by Dingguo Biotechnology (Beijing, China). *PrimeSTAR* Max DNA polymerase, *PrimeSTAR* HS DNA polymerase, *rTaq* DNA polymerase, QuickCut^™^ *Dpn*I were purchased from Takara (Dalian, China). Plasmid extraction, DNA gel purification kits and all primers were supplied by Sangon Biotech (Shanghai, China). The primary antibody (anti-2A peptide) was purchased from Millipore (USA). Agarose, BSA and HRP-labeled goat anti-mouse IgG (H + L) were purchased from Beyotime (Shanghai, China). All other chemicals were purchased from Bonuo Biological and Chemical Reagent Company (Dalian, China).

### Plasmid construction, transformation, and verification

The gene of *MAN5C* originated from *P. lilacinus* ATCC 36010 (Sugino et al. 2004), was codon-optimized according to the codon preference of *R. toruloides*, and synthesized by Synbio Technologies (Suzhou, China). The optimized gene sequences are listed in Additional file 1: Table S1. To construct the plasmid pZPK-P_*ADH2*_-*MAN5C*-P2A-*HYG*-T_*HSP*_, the fragment *MAN5C* was firstly amplified from plasmid pUC-*MAN5C* by the primer pair PGK-MAN5C-F and MAN5C(his)-P2A-R, and then cloned into the pZPK-P_*PGK*_-*MNP*-P2A-*HYG*-T_*HSP*_ (Lyu et al. 2021) by the restriction-free (RF) cloning procedure (Unger et al. 2010). Secondly, the primer pair PZPK-ADH2-F and ADH2-MAN5C-R were used to amplify P_*ADH2*_ fragment from plasmid pZPK-PADH2, which was then substituted for P_*PGK*_ to give pZPK-P_*ADH2*_-*MAN5C*-P2A-*HYG*-T_*HSP*_. All plasmids and primers used in this study are listed in Table 1 and Additional file 1: Table S2, respectively.

The plasmid pZPK-P_*ADH2*_-*MAN5C*-P2A-*HYG*-T_*HSP*_ was amplified in *E. coli* DH10B, transformed into *A. tumefaciens* AGL1, and then used to transform *R. toruloides* NP11 according to *Agrobacterium*-mediated transformation method as described previously (Lin et al. 2014). The transformants were resuspended in sterile water and spread on YEPD plates supplemented with 50 μg/mL hygromycin and 300 μg/mL cefotaxime to recover single clones. To verify proper integration of the expected DNA fragment, *R. toruloides* colonies were subjected to colony-PCR with the primer pair of MAN5C-NF and MAN5C-NR according to previously described method (Lin et al. 2012).

### Western blot

Western blot analysis was used to further confirm whether the MAN5C protein had been successfully expressed and secreted into the medium by *R. toruloides* transformants. Sample preparation and experiment operation was carried out as described above with some modification (Jiao et al. 2018; Liu et al. 2019). The cells were harvested in the early stationary phase by centrifugation at 9000 × *g* for 3 min and resuspended in 400 μL lysis buffer, and then homogenized by FastPrep instrument (MP biomedicals, USA) for 5 cycles at a speed of 4.0 m/s for 45 s each, with ice-water bath for 3 min of interval. Cell lysates were collected after centrifuging at 13000 × *g* for 5 min. To detect whether MAN5C were secreted outside the cells, the supernatants of broth were sampled after culturing for 54 h.

Proteins were separated by 12% SDS-polyacrylamide and then transferred onto nitrocellulose membranes (Pall Corporation, Pensacola, USA). The membranes were incubated with the primary antibody anti-2A peptide and the secondary antibody HRP-labeled goat anti-mouse IgG (H + L) after blocking with BSA and washing. The western blot results were visualized by soaking in Tanon High-sig ECL western blotting substrate (Tanon, Shanghai, China), and photographed by Tanon system (GelCap).

### Growth curve determination

Cells were cultured in 5 mL YEPD medium for 24 h to prepare seeds and then measured the cell density at 600 nm (OD_600_) using a spectrophotometer (Evolution 220; Thermo Fisher Scientific, USA). Seed cultures were added to 1 mL YEPD medium in 48-well plates at an initial OD_600_ of 0.2 and cultivated at 30 ℃, 800 rpm by a high throughput microbial growth curve analysis system (Jieling, Tianjin, China), which measured the absorbance at 600 nm every 30 min. The specific growth rate and lag phase data were estimated from absorbance growth curves using the Schnute model (Zwietering et al. 1990).

### Cell wall integrity analysis

Congo red (CR) susceptibility test was used to evaluate the cell wall integrity of engineered strains (Ram and Klis 2006). Briefly, *R. toruloides* cell cultures were sequentially diluted and spotted on YEPD agar plates and those containing 150 μg/mL CR, incubated at 30 ℃ for 3–4 days. Cell growth profiles of the plates were recorded with a camera.

### Culture conditions

*R. toruloides* cells were pre-cultured in 50 mL YEPD medium for 24 h as seed cultures. Lipid production was conducted at 30 ℃, 200 rpm in a 500-mL Erlenmeyer flask containing 100 mL of nitrogen-limited medium that was inoculated with 10% seed culture. The lipid production broth was sampled to measure OD_600_ and residual glucose every 24 h by a spectrophotometer (Evolution 220; Thermo Fisher Scientific, USA) and a glucose analyzer (SBA-50B; Shandong Academy of Sciences, Jinan, China), respectively. At the same time, cells and supernatants of the broth were collected to prepare protein samples for analysis. All cultures were done in triplicates.

### MAN5C activity assay

The activity of MAN5C was assayed according to an established procedure (Yang et al. 2011). One unit of MAN5C activity was defined as the volume of the sample solution which liberated reducing sugar equivalent to 10 μg mannose per minute at 37 ℃, pH 4.5.

### Lipid extraction procedure

At the end of the lipid production experiment, the culture broth was divided into 5 parts of 15 mL each for different purposes. Of which 5 mL of each sample was prepared for environmental scanning electron microscopy (ESEM) observation. Wet cells of the first part were collected by centrifugation at 9190 × *g* for 5 min, washed with 10 mL of distilled water twice, and dried at 105 ℃ to a constant weight, which was used to determine DCW gravimetrically and estimate total lipids as described previously (Li et al. 2007).

The second part was used to extract lipids with no other treatment (Method I). The third part was treated in a boiling water bath for 20 min followed by incubation at 37 ℃, 200 rpm for 2 h (Method II). For the fourth part, wet cells were separated and resuspended in 10 mL water, held in a boiling water bath for 20 min, centrifuged and resuspended in the supernatants of the culture broth, followed by incubation at 37 ℃, 200 rpm for 2 h (Method III). The fifth part was held in a water bath at 50 ℃, 200 rpm for 24 h (Method IV). Lipid extraction of the second to fifth part was performed as previously described with slight modifications (Jin et al. 2012). Briefly, the extraction of the broth was carried out with 10 mL ethyl acetate at 30 ℃, 200 rpm for 1 h. The organic phase was collected by centrifugation at 9190 × g for 5 min, and 10 mL of ethyl acetate was added to the broth to extract again by vortex for 1 min. The organic phases were combined, washed with an equal volume of 0.1% sodium chloride, separated by centrifugation at 9190 × *g* for 5 min, and dried with anhydrous sodium sulfate. Total lipids were obtained upon organic solvents removed by rotary evaporation and dried at 105 ℃ to a constant weight.

### Environmental scanning electron microscopy analysis

The cell morphology of different samples was directly imaged by Quanta FEG 650 ESEM (FEI Ltd., USA) at 200 Pa, 5 ℃ or −5 ℃ with magnification of 20000 × .

### Analytical methods

All assays were done in triplicates, and the results were expressed as mean values and standard deviations. The statistical significance analysis was performed by Student’s *t*-test (*, *P* < 0.05; **, *P* < 0.01; ***, *P* < 0.001). Lipid content and lipid yield was expressed as gram of lipids per gram of DCW and consumed glucose. To quantify the lipid extraction efficiency of different methods, lipid extraction yield (LEY) was defined as follow:

$${\text{Lipid}}\,{\text{extraction}}\,{\text{yield}}\,\left( \% \right)\,{ = }\,{\text{lipids/total}}\,{\text{lipids}}\, \times \,{\text{100\% }}$$

## Results

### Secretory expression of MAN5C in R. toruloides

**Fig. 2:**
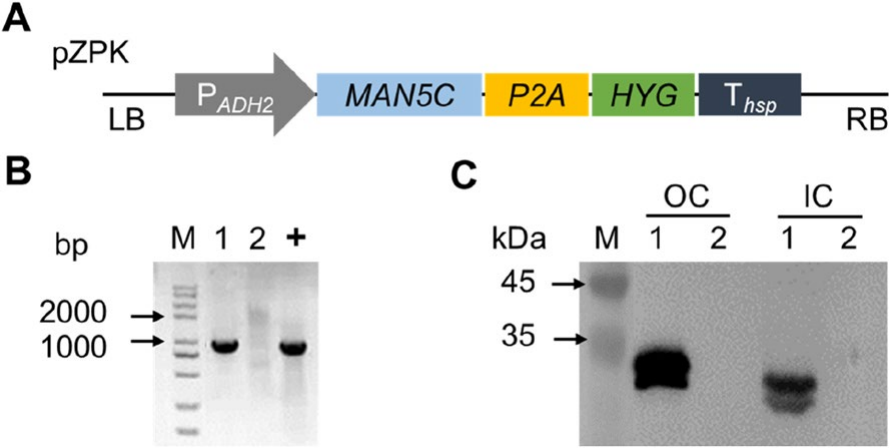
Engineering *R. toruloides* NP11 for MAN5C secretory expression. **A** Schematic of MAN5C expression cassette; **B** PCR result for *MAN5C* gene verification using the genomic DNA as templates; **C** Western blot analysis of the recombinant MAN5C. Lane M: Marker; Lane 1, 2: Represent samples from NP11-MAN5C and NP11, respectively; Lane + : The plasmid pZPK-P_*ADH2*_-*MAN5C*-P2A-*HYG*-T_*HSP*_; OC, IC: Represent the protein samples from the culture supernatant and cell lysates, respectively

The *MAN5C* gene cassette under the promoter P_*ADH2*_ (Fig. 2A) was constructed and integrated into the genome of *R. toruloides* NP11 to obtain the recombinant strain NP11-MAN5C. It should be noted that *MAN5C* naturally encodes a signal peptide of 21 amino acids at its N-terminus. Furthermore, recombinant MAN5C was fused with a His_6_-tag to the C-terminus and 2A peptide sequence. Colony PCR with primer pair MAN5C-NF/MAN5C-NR showed a DNA fragment of 1.0 kb as expected, confirming the integration of exogenous gene in NP11-MAN5C genome (Fig. 2B). According to the self-cleaving mechanism of P2A-peptide, proper expression of MAN5C should be verified by detection of the P2A-peptide through Western blot assay. To confirm successful expression of MAN5C, samples of the culture supernatant and lysates of NP11-MAN5C cells were examined. Two very close bands appeared slightly below 35 kDa for samples from NP11-MAN5C, and no bands were found for those from the parental strain NP11 (Fig. 2C), suggesting that MAN5C was expressed and secreted by the engineered strain. The mass differences between these two bands were likely caused by partial removal of the signal peptide. It should be noted that similar phenomena have been demonstrated for secretory expression of green fluorescent protein in *Pichia pastoris* (Huang et al. 2018).

### Cell growth and lipid production profiles

**Fig. 3:**
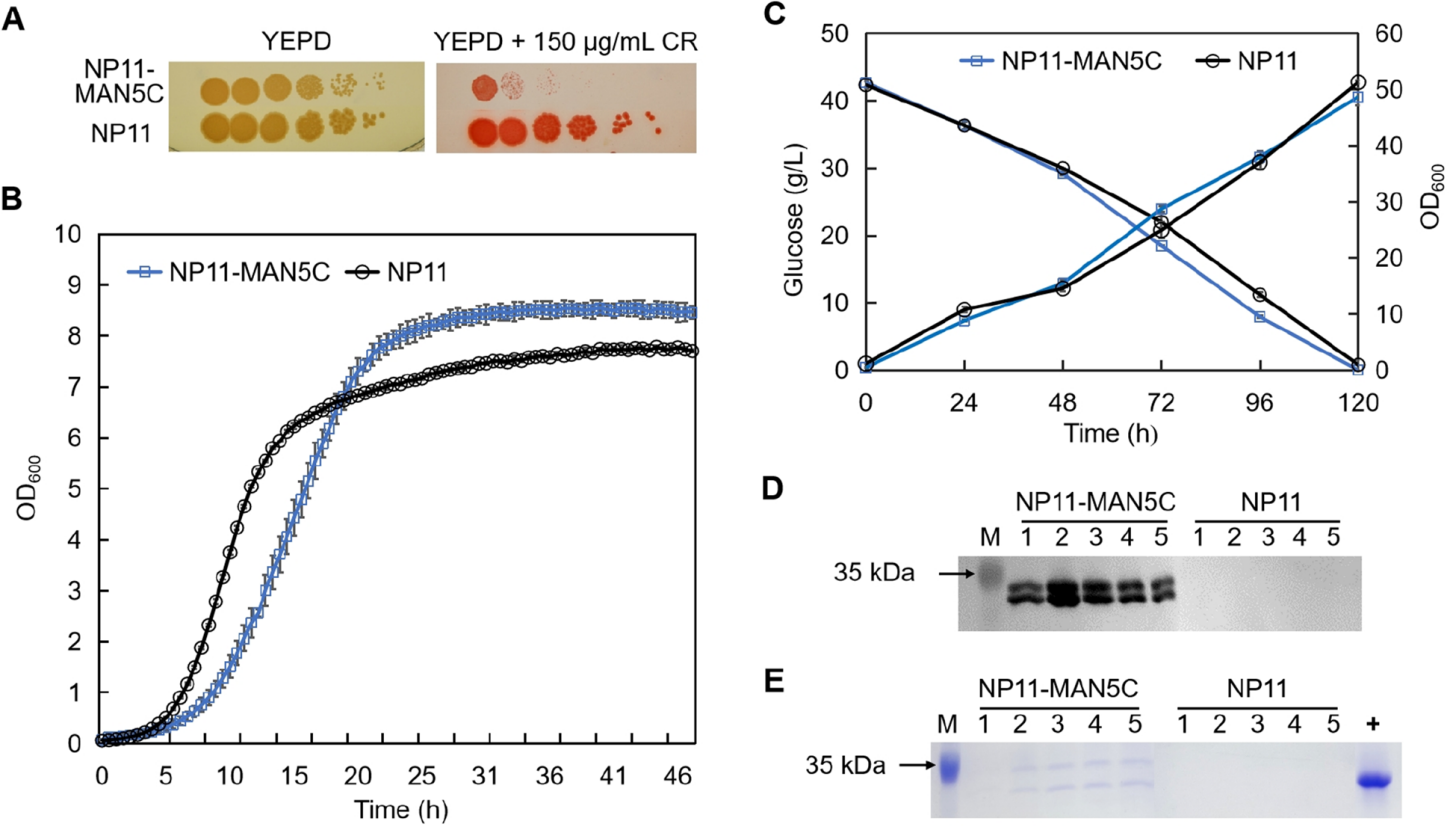
Results of cell growth and MAN5C production. **A** Cell growth profiles on YEPD agar plate and that supplemented with Congo red; **B** Cell growth profiles in YEPD media at 30 ℃, 800 rpm in a microbial growth analysis system; **C** Profiles of glucose consumption and cell growth in nitrogen-limited media at 30 ℃, 200 rpm; **D** Western blot results of the supernatants of cell lysates; **E** SDS-PAGE results of the culture broth. Lane M: Marker; Lanes 1 to 5 were samples from the culture in nitrogen-limited media at 24, 48, 72, 96, and 120 h, respectively; Lane + : Mature MAN5C expressed by *P. pastoris* X-33

To evaluate the biological effects of MAN5C expression in *R. toruloides*, we grew cells on YEPD agar plates in the presence of CR. It was found that the growth of NP11-MAN5C cells was significantly inhibited by CR, but NP11 cells showed similar profiles to those on agar plate (Fig. 3A). The fact that NP11-MAN5C cells were sensitive to CR indicated that MAN5C led to reduced cell wall integrity (Ram and Klis 2006). Next, NP11-MAN5C cells were grown in YEPD medium and cell density was monitored. It was found that NP11-MAN5C and NP11 (control) had similar lag-phase, but NP11-MAN5C cells grew much slower (Fig. 3B, Table 2). These results indicated that expression of MAN5C had limited effects on cell growth in nutrient rich media.

**Table 2:** Results of cell cultures in different media

Strains	YEPD media		Nitrogen-limited media			
	Growth rate (h^−1^)	Lag phase (h)	DCW (g/L)	Lipid (g/L)	Lipid content (%)	Lipid yield (g/g)
NP11-MAN5C	0.35 ± 0.01	1.43 ± 0.12	11.8 ± 0.1	6.6 ± 0.0	55.7 ± 0.4	0.15 ± 0.00
NP11	0.57 ± 0.01	1.14 ± 0.08	12.0 ± 0.2	5.6 ± 0.4	46.4 ± 3.2	0.13 ± 0.01

Next, lipid production performance was evaluated by cultivating NP11-MAN5C cells in nitrogen-limited media. It was found that the profiles of glucose consumption and cell density evolution of NP11-MAN5C cells were almost identical to those of NP11 cells (Fig. 3C). Thus, these two strains afforded very close DCW data (Table 2). However, the engineered strain NP11-MAN5C showed a significantly higher lipid content, leading to apparent higher lipid titer and lipid yield than those of NP11. A plausible explanation was that the presence of MAN5C might prevent cells from accumulating excessive glucomannan within the cell wall, thus saving more carbon source for improved lipid production.

The expression and distribution of MAN5C were also monitored over time during the lipid production process. Samples of the supernatants of cell lysates and the culture broth were subjected to Western blot and SDS-PAGE analysis, respectively. It was found that Western blot results showed two separated bands below the 35-kDa marker band throughout the cultivation of the NP11-MAN5C (Fig. 3D). This was in agreement with the results observed under nutrient-rich media (Fig. 2C), suggesting a proper expression of MAN5C under nitrogen-limited condition. Moreover, similar bands were detected from the culture broth of NP11-MAN5C by SDS-PAGE analysis (Fig. 3E). It is worth mentioning that the size differences between the two bands is about 2 kDa, which is consistent with the calculate mass of signal peptide (Sugino et al. 2004). Thus, it is reasonable to infer that the higher bands might be the full length of MAN5C without signal peptide removal. Notably, a band at around 33 kDa was shown for the positive control corresponding to the mature MAN5C secreted by engineered *Pichia pastoris* X-33 cells (Yang et al. 2011). To confirm the presence of active MAN5C in the lipid production culture broth, supernatants of the culture at the endpoint were sampled and assayed. It was found that MAN5C activity was 5.1 U/mL, indicating that NP11-MAN5C cells were able to produce and secret active MAN5C.

### Lipid extraction process development

**Fig. 4:**
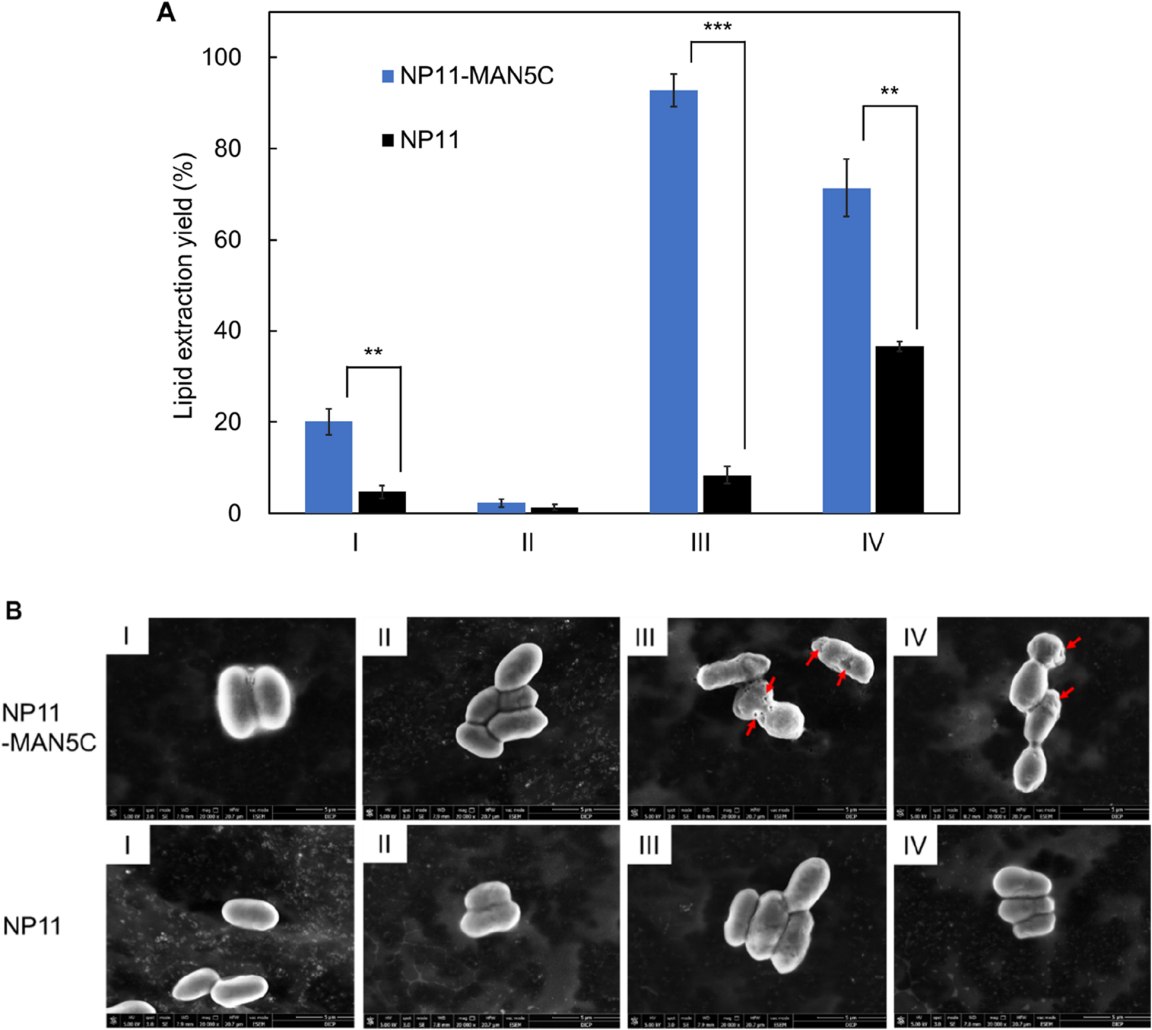
Lipid extraction yields and cell morphology with different methods. **A** Lipid extraction yields. Cells were treated according to different methods and then extracted with ethyl acetate at 30 ℃. **B** ESEM analysis of *R. toruloides* cells being treated with different methods. **Method I.** With no treatment; **Method II.** Heated in a boiling water bath for 20 min; **Method III.** Cells were separated, resuspended in water, heated in a boiling water bath for 20 min, separated and then resuspended in the supernatant of the corresponding culture broth; **Method IV.** Heated in a water bath at 50 ℃ for 24 h

To demonstrate whether secretory expression of MAN5C facilitated lipid extraction, different processes were evaluated with lipid production culture of NP11-MAN5C, and lipid extraction yield (LEY) was estimated. As shown in Fig. 4A, when NP11-MAN5C cell cultures were extract with ethyl acetate directly, the LEY was 20.1%, fourfold higher than that of NP11 (Method I). When the cultures were treated in boiling water bath, the LEY of two strains were below 2.5% with no significant differences (Method II). These results indicated that heat treatment had detrimental effects for lipid extraction, likely by deactivating cell lytic enzymes including MAN5C. Earlier studies showed that alive *R. toruloides* cells were resistant to lysis by MAN5C, however, heat treatment made cells more susceptible to enzymatic hydrolysis (Jin et al. 2012; Murao et al. 1976). Therefore, cells were separated, heated in boiling water bath, then resuspended in the culture supernatant, and finally extracted with ethyl acetate at 30 ℃ (Method III). As a result, the LEY of NP11-MAN5C reached 92.8%, 11-fold higher than that of NP11, indicating that heat treatment greatly improved cell wall digestibility and that the amount of active MAN5C present in the culture supernatant was sufficient to loosen cell wall for lipid extraction. More interestingly, these results also suggested that the cell wall integrity of NP11-MAN5C was reduced comparing to that of NP11. Considering that the optimal temperature for MAN5C was at 50 ℃ (Yang et al. 2011), the cell cultures were treated in a water bath at 50 ℃ for 24 h followed by lipid extraction (Method IV), and the LEY of NP11-MAN5C was 71.4%, about twofold higher than that of NP11. These results were in well agreement with the presence of active MAN5C in the culture broth of NP11-MAN5C. Overall, our results showed that MAN5C could play a major role to assist lipid extraction in the presence of ethyl acetate when cells were deactivated by heating (Method III and IV).

In parallel, *R. toruloides* cells being treated with different methods were also observed with the ESEM technology, and the results were shown in Fig. 4B. It was clear that surfaces of NP11-MAN5C and the NP11 cells were smooth and complete regardless of being alive or deactivated upon heat-treatment in boiling water bath. The results supported the fact that lipids were barely extractable from these samples (*vide anti*). Also, it indicated that secretory expression of MAN5C in *R. toruloides* had little effects on cell morphology. In contrast, when NP11-MAN5C cells were heat-treated shortly in boiling water bath followed by incubation in the supernatant of the cell culture (Method III), or were treated at 50 ℃ for 24 h (Method IV), cells became fragile and had to be photographed at −5 ℃. Remarkably, holes and defects were clearly observed on the surfaces of these samples, which correlated very well with lipid extraction results.

## Discussion

While it has been emerged as a promising host for the accumulation of lipids and terpenoids, the basidiomycetous yeast *R. toruloides* has relatively tight cell wall with glucomannan as a major structure component that hinders the extraction of intracellular products. To facilitate product extraction from yeast cells, different cell disruption methods have been used in the literature as summarized in a recent review (Zainuddin et al. 2021). While mechanical methods such as ultrasonication and homogenization require additional equipment, nonmechanical methods such as enzymatic or chemical digestion of yeast cells are more convenient. Enzymatic lysis of cell wall is desirable over other methods as it can be operated under mild conditions for efficient recovery of lipids and spent cell mass. Thus, a recombinant MAN5C has been applied successfully in lipid extraction from *R. toruloides* cells with high lipid recovery yield (Jin et al. 2012). However, the costs of using purified MAN5C would be high especially once the process scaled up. Inspired by an early study that a heterologous expression of bacterial 3-dehydroshikimate dehydratase in *Arabidopsis* reduced lignin content of the cell wall leading to improved saccharification efficiency (Eudes et al. 2015), we integrated an *MAN5C* cassette into the genome of *R. toruloides* NP11 for secretory expression of MAN5C. It was expected that the recombinant strain might have weaker cell wall in favor of lipid extraction. Indeed, when the culture broth was extracted with ethyl acetate directly, up to 20% of total lipids were recovered, which was significantly improved comparing with that of the parental strain. When NP11-MAN5C cells or the corresponding culture broth were subject to heat treatment followed by lipid extraction in the presence of MAN5C produced in situ, up to 92% of total lipids were received. Compared with the previous studies (Jin et al. 2012; Kruger et al. 2018), NP11-MAN5C cells produce MAN5C during the culture process, which can greatly reduce the costs of lipid extraction by avoiding the usage of purified enzymes. Moreover, this strategy is green and equipment friendly because no extra acids or bases is used to assist lipid extraction. It should be noted that we also constructed the *MAN5C* cassette with the P_PGK_ promoter to drive protein expression, and found NP11 transformants gave quite similar lipid extraction promotion effects according to the procedures of Method III (data not shown). Similarly, other enzymes such as glucanase, chitinase and pectinase may be evaluated by in virto applications as did previously (Jin et al. 2012), and then explored to further manipulate the cell wall integrity of oleaginous yeasts and improve the efficiency of lipid extraction.

In another scenario, this is the first report that the oleaginous yeast *R. toruloides* was engineered to secretory expression of heterologous protein fused with exogenous signal peptide. Recently, there are some studies showing that signal peptides could be predicted from secretion profiles and in-silico analysis (Lebre et al. 2018; Massahi and Calik 2015). Thus, endogenous signal peptides related to protein translocation may be revealed by analysis of the multi-omic data of *R. toruloides* (Zhu et al. 2012), and applied to strain engineering. Furthermore, genes involving cell wall biosynthesis may be targeted to reduce cell wall integrity and improve protein secretion (Li et al. 2020; Naranjo et al. 2019). In the future, these strategies should be explored to devise advanced *R. toruloides* strains for more effective lipid recovery but little compromise in terms of overall lipid production capacity.

## Conclusion

In the present study, *R. toruloides* NP11 was engineered to secretory expression of MAN5C from the fungi *P. lilacinus* ATCC 36010 for improved lipid extraction. It was found that the engineered strain NP11-MAN5C could secrete active MAN5C into the culture broth yet produce lipids with slightly improved lipid production profiles. The in-situ produced MAN5C facilitated new downstream processes for efficient lipid extraction from wet *R. toruloides* cells.

This study provides a new strategy to engineer oleaginous yeasts for more cost-effective lipid extraction.

### Supplementary Information

**Additional file 1: Table S1.**The optimized gene sequence encoding MAN5C. **Table S2.** Primers and their sequences used in this study.

## Data Availability

All data sets used and analyzed are available on reasonable request.
