# Supplementary material for: Secretory expression of β-1,3-glucomannanase in the oleaginous yeast Rhodosporidium toruloides for improved lipid extraction
Source: Bioresour Bioprocess. 2023 Mar 2;10(1):16. doi: 10.1186/s40643-023-00639-2 (PMC10991151; doi:10.1186/s40643-023-00639-2)
Supplement: Supplementary file 1 — Additional file 1: Table S1.The optimized gene sequence encoding MAN5C. Table S2. Primers and their sequences used in this study. [file 40643_2023_639_MOESM1_ESM.docx]

**Secretory Expression of β-1,3-Glucomannanase in the Oleaginous Yeast *Rhodosporidium toruloides*** **for Improved Lipid Extraction**

Shiyu Liang^1,2^, Yue Zhang^1,2^, Liting Lyu^1^, Shuang Wang^1,2^, Zongbao K. Zhao^1*^

Table S1. The optimized gene sequence encoding MAN5C.

| ATGCGCTTCTACTCGATCATCTACCAGGCCCTCGCTCTCGCTTCGCTCCCTCTCGCTCTCGCCGCTAACTCGTTCTCGGCCTCGAACCTCTACTACGCCGCCGGACTCACCGTCGACCAGCAGCACACCCTCCTCTCGGGACTCCAGTCGGCCGGAGTCAAGGTCCTCCGCGTCTGGCTCGACGGACAGTCGGGAAACGTCAAGGGCACCCCGATCAACGGCTTCAACGGCCTCCAGGGAGCTTCGCCAGACTCGTGGGACGACACCGTCCTCAACCGCCTCGACGACTTCATGTTCAACGCCCACTCGTACGGCATCAAGCTCCTCGTCTCGATCCACTCGTACAACGCCCTCGCCGCCAACGCCGACTTCTACGGCAAGTGGTACGGCACCGGCGACTTCTACACCAACGGCGACGCCATCAACTACTTCAAGCAGCGCATCGCCCACGTCCTCGCCCACGTCAACCCGCACAACAACAAGCCTTGGTCGCAGTCGTCGGAGTACATCTTCGCCTTCGAGGCCCAGAACGAGGCCATGCACGACCAGGAGAACCCGTCGGCTCTCCAGTCGTGGCAGTGCACGATGGCCCAGGCCATCAAGGACAACCTCAAGGGCTCGACCGCCATCCTCGTCTCGACCGGAGGAGGAGCTTGGCTCGCCAACTCGCTCCTCGACGGCTACTTCTCGTGCTCGGCCCTCGACGTCCTCGCTATCCACGCCTACGGAACCGGCGACTTCGCTACCTCGGCCCTCAAGCCTTACGTCACCCGCGCTCAGCAGGCCGGCAAGAAGCTCATCATGCAGGAGTGGGGCGCTTGCTACACCACCGCCTCGAACAACAACTGCAACGGCGGCTCGCCGCTCAACTCGGGCACCCGCGACAACAACATCAAGTCGTGGGCCGCTTCGATCACCGCCGCCGGAATCCCTTGGTTCTACTGGCAGATCCTCCCGAACGAGGACCCTCACCAGGGCTGGGACTACGAGGTCGGCATCAACGGCGTCAATTGGGGCGCTCTCCAGACCGCCGGACTCGCCGCCGGAAAGGCTCAGGCCGCCTTCGACTTCTCGAAGTGGCTCCTCCACCACCACCACCACCAC |
| --- |

Table S2. Primers and their sequences used in this study.

| Primer name | Sequence (5 ʹ - 3ʹ) |
| --- | --- |
| PGK-MAN5C-F | GCAGGTTCACAGCAACTCACCCGTCCAACTCCCACCCTCCCCCGTGCAGCCCACCATGCGCTTCTACTCGATCATCTAC |
| MAN5C(his)-P2A-R | GCCAGGGTTCTCTTCGACGTCGCCAGCCTGCTTGAGGAGCGAGAAGTTGGTAGCGCCCGAGCCGTGGTGGTGGTGGTGGTGGAGGAGC |
| PZPK-ADH2-F | CGGACGTTTTTAATGTACTGAATTAACGCCGAATTGAATTCGAGCTCGGTACCCGGGGGCTGAGGCTTCCCCGACGCCCCTC |
| ADH2-MAN5C-R | GCGAGAGGGAGCGAAGCGAGAGCGAGGGCCTGGTAGATGATCGAGTAGAAGCGCATTGTGACTGTCGGAGACGTGGCAGC |
| PGK-F | CGCATCGTTGAACTTGCACTTC |
| HYG-R | CGAGACCGAGTCGAACTTTTCGATG |
| MAN5C-NF | CGCCGCTAACTCGTTCTCG |
| MAN5C-NR | CTGGAGAGCGCCCCAATTG |
| ADH2-115-F | GGGAGCTGTTGGCTGGCTGG |
| ADH2-110-R | GGGTGTGCTGCTGGTCGACG |
| GAPDH-F | GGTATCGCCCTCAACGACA |
| GAPDH-R | GACGAGCAAGTCCACGACAC |
